# Around the Hospitals

**Published:** 1893-07-22

**Authors:** 


### Around the Hospitals.

Notice to the Managers of Institutions.—Special space is now reserved for the insertion of notices of hospital meetings and festivals. The Editor requests that all notices may reach The Hospital Office, 428, Strand, at least one week before the date of each meeting.

**Hospital for Sick Children, Toronto.**—The members of the Ontario Medical Association were lately invited by the Board of Trustees of this hospital to pay a visit of inspection. A thorough examination into the details and working of the institution drew forth expressions of unqualified approval from the many doctors who availed themselves of the invitation. The architectural and sanitary arrangements, and those for ventilation and heating, won hearty commendation from the visitors, who minutely inspected every portion of the building. After ample justice had been done to an excellent luncheon the guests collected in the main ward, while an address of welcome was read by the Chairman, Mr. J. Ross Robertson, on behalf of the Trustees of the Hospital. Glancing at the past history of the institution, Mr. Robertson said that twenty years ago no hospital for sick children existed in the Dominion. Then the efforts of a few earnest women resulted in the commencement, on a small scale, of a movement which has now developed into a splendid building, "equipped with all the appointments necessary to cure or relieve those little ones who come under its care." Mr. Robertson hoped all were satisfied with what they had seen and would continue to do what they could to help in still further improving the general arrangements. Dr. Harrison, Dr. McKay, and Dr. Thorburn severally responded for the visitors, and expressed their warm sympathy with the work of the hospital and their appreciation of its excellent management. There were not many patients in the hospital at this time, most of the little ones having been sent for the summer to Toronto Island, where is the "Lakeside Home" in connexion with the hospital. Some eighty of the invalid children were established here, and the members of the Medical Association paid the Home a visit on the day following their inspection of the hospital in College Street. Here also existing arrangements were unanimously approved, and the Trustees were deservedly congratulated upon the success which was attending both institutions, success which it is evidently the intention of the authorities shall continue, their object being in Mr. Robertson's own words, "to make this hospital so perfect that when in any part of the world they want to erect such buildings they must come to Toronto to learn how it is done."

